# Isometric scaling of hibernation: when trees do not let the forest be seen

**DOI:** 10.1098/rspb.2022.1719

**Published:** 2022-10-12

**Authors:** Roberto F. Nespolo, Carlos Mejias, Francisco Bozinovic

**Affiliations:** ^1^ Instituto de Ciencias Ambientales y Evolutivas, Universidad Austral de Chile, Valdivia, Los Ríos, Chile; ^2^ Facultad de Ciencias, Universidad Austral de Chile, Valdivia, Los Ríos, Chile; ^3^ Magister en Ecología Aplicada, Facultad de Ciencias, Universidad Austral de Chile, Valdivia, Los Ríos, Chile; ^4^ Center of Applied Ecology and Sustainability (CAPES), Pontificia Universidad Católica de Chile, Santiago, Chile; ^5^ Departamento de Ecología Facultad de Ciencias Biológicas, Pontificia Universidad Católica de Chile, Santiago, Chile; ^6^ Millennium Nucleus of Patagonian Limit of Life (LiLi) and iBio Millennium Institute, Santiago, Chile

Mammalian hibernation has fascinated scientists for more than a century [1], but surprisingly, few attempts have been done to estimate the actual amount of energy that is saved by hibernation. In a previous article [2], we compared daily energy expenditure of hibernation (DEE_H_) with the predicted basal metabolic rate (BMR) of several hibernators, to calculate savings at different body masses (*M*_B_). Our main finding was that DEE_H_ relates isometrically with mass, which we interpreted in terms of the existence of a minimum cellular metabolism (see Discussion in [2]). Another implication of our work is the fact that the theoretical size predicted by us, at which the curve of DEE_H_ and BMR crosses (i.e. when energy savings by hibernation becomes zero; hereafter M_B_zero), is lower (= 75 kg, using BMR = 2.06*M*_B_^0.67^ from [3]) than previous estimations (15–115 tons, see [4,5]). We also discussed the fact that M_B_zero falls within the range of present-day hibernators (i.e., black and brown bears). Additionally, we calculated M_B_zero using daily energy expenditure (DEE), which gave 1549.7 kg, but we argued that the use of BMR is more appropriate, because a torpid animal is in a resting state, in which the only difference from an active animal is that it is not paying the endothermic costs. Then, in a comment published in this issue, Tøien *et al*. [6] questioned our conclusions arguing that (i) empirical BMR values should be used, instead of allometric predictions; (ii) some of the data (e.g. for bears) considered lactating females or animals that probably had access to food during hibernation (e.g. arctic ground squirrels); and (iii) using an allometric exponent of 0.67 for BMR is inappropriate because it was obtained after Q_10_ corrections.^1^ They also cited two studies were oxygen consumption was measured in hibernating black bears, showing a metabolic reduction to 25% of BMR when torpid (i.e. they save 75%) [7,8]. Finally, using empirical values of BMR and a corrected sample, they estimated M_B_zero = 2250 kg. Beyond discussing whether bears save energy when hibernating (they certainly do), we reanalysed the datasets to give some perspective to readers. Here we show (i) that our original conclusions were correct, (ii) that small deviations of single datapoints do not affect the regression analysis and (iii) that mass-specific units distort any interpretation.

Given that hibernators vary their energy consumption depending on environmental temperature, and reduce their metabolism as the cold season progresses [9,10], we argued that averaged energy consumption during long periods is a better measure than instantaneous measures of metabolic rates (the most common practice). What could make a difference, however, is the cost of arousal, which can contribute 21% to the total hibernation energy expenditure (see electronic supplementary material). In addition, different species experience different arousal frequencies during hibernation. Then, we considered those costs and frequencies for generating a dataset corrected by arousal costs, to be explored (DEE_H_-arousal, see electronic supplementary material).

We re-analysed the datasets of Nespolo *et al*. and Tøien *et al*. using the online spreadsheet provided by the later authors (table 1; see also an R script provided in electronic supplementary material). We used three BMR equations: the original equation of White & Seymour [3] (exponent: 0.67), who compiled BMR for 507 species, but data were corrected using the Q_10_ factor; the equation of McNab [11] (exponent: 0.72), which included BMR for 639 species, and also considered the most complete available dataset [12]; and the empirical equation provided by Tøien *et al*. for 15 species (exponent: 0.80).

**Table 1.** Reanalysis of Tøien *et al*. [6] and Nespolo *et al*. [2] datasets for DEE_H_ (kJd^−1^), and different intersection points estimated for the curves of DEE_H_ and BMR (kJd^−1^), predicted by three different allometric equations. This intersection represents the theoretical body mass where energy savings of hibernation become zero (M_B_zero). DEE_H_ was obtained from mass reduction after hibernation in different species (see details in [2]). DEE_H_-arousals represents the value after correcting by periodic arousals (details in the electronic supplementary material). All the parameters in this table are reproduced by an R script provided upon request and also provided in the electronic supplementary material. In all cases, regressions were highly significant with *R*^2^ values above 0.94.

variable	dataset	log–log regression results^a^			body mass (kg) at the intersection (M_B_zero)^b^		
		log10(***a***)	***a*** (intercept)	***b*** (exponent)	BMR = 2.062*M*_B_^0.67^ (White & Seymour [3], *n* = 507)^c^	BMR = 1.68*M*_B_^0.72^ (McNab [11], *n* = 639)^d^	BMR = 1.09*M*_B_^0.80^ (Tøien *et al*. [6], *n* = 15)
DEE_H_	NespoloEA	−1.126	0.075	0.968	67.832	293.994	7106.096
DEE_H_-_arousals_	NespoloEA	−1.398	0.040	1.022	72.506	244.265	2568.875
DEE_H_	TøienEA	−1.086	0.082	0.952	93.318	481.905	21006.799
DEE_H_-_arousals_	TøienEA	−1.360	0.044	1.007	92.329	345.992	4894.727

^a^The equation of a log–log linear regression is: log_10_(*Y*) = log_10_(*a*) + *b*log_10_(*X*), equivalent to: *Y* = *aX^b^*.

^b^Calculating body mass (*M*_B_) at the intersection point requires to equate *aM*_B_*^b^* = *cM*_B_*^d^,* which solving *M*_B_ gives *M*_B_ = 10^[log_10_^^(*a*)/log_10_^^(*c*)]/(*d –* *b*)^.

^c^Equation of Fig. 2d in the original publication: BMR = 4.34 *M*_B_^0.67^ (mlO_2_ h^–^^1^), converted to kJd^-1^ using an RQ of 0.71.

^d^Equation (1) in the original publication: BMR = 0.070 *M*_B_^0.721^ (kJ h^−1^) converted to kJd^−1^ by multiplying by 24.

All log–log regressions, either including bears (Nespolo *et al*. dataset), or not (Tøien *et al*. dataset), or correcting by arousals (or not), generated an exponent of 1.0, when expressed with one decimal (table 1). This is a support to our original conclusion. Contrarily to regressions, point estimations are highly sensitive to small deviations of the data, thus we used analytical solutions and three decimals for estimating the cross point between all the combinations of equations and datasets. Calculating M_B_zero requires solving *M*_B_ in: *aM_B_^b^* = *cM_B_^d^*, which gives *M*_B_ = 10^[log_10_^^(*a*)/log_10_^^(*c*)]/(*d*-*b*)^. Thus, using the White & Seymour equation M_B_zero ranges from 68 to 92 kg (as we originally reported; mean = 81.5, s.e. = 6.6 kg; sixth column in table 1). Using McNab's equation, mean M_B_zero is 341.5 kg (s.e. = 51.2 kg; 244.3–481.9 kg). and using Tøien's equation (from empirical data), this number is 8894.1 kg (s.e. = 4141.4 kg; range: 2568.9–21 006.8 kg; table 1, last column). These large s.e. and variation confirms the great sensitivity to single datapoints of small samples, which is the case for the later dataset. This is why we think it is not correct to use few empirical data if allometric equations are available: allometric equations are global generalizations of hundreds of species. Ignoring Tøien's equation, and also the criticized Q_10_-corrected equation of White & Seymour [3], leaves us with McNab's allometric predictions, which raise M_B_zero from tens of kg (our previous estimate) to a few hundred (this work).

Tøien *et al*. [6] provided an alternative biological explanation for the isometric scaling of hibernation. They suggested that ‘storage of fat and other substrates used as energy sources during hibernation is limited by body volume and scales isometrically with body mass, and thus DEE_H_ will also scale near isometrically with body mass. Since mass-specific BMR increases exponentially with decreasing body mass in mammals, energy savings during hibernation will also increase exponentially as body mass decreases, and this is affected by active suppression of metabolism and decreasing body temperature.’

We did not find support to these assertions in our copy of their paper. We only found a second plot in their fig. 1 (right panel), without regression statistics, where log(mass-specific metabolism) is plotted against log(*M*_B_)(we refer later to the problems of mass-specific units). The legend of this figure reads: ‘Right panel: same data and regression lines expressed as kJ day^−1^ kg^−1^ on linear *y*-axis versus BM(g) expressed on logarithmic *x*-axis, showing the exponential increase in mass-specific BMR and need to save energy with decreasing body mass below 2268 kg, while mass-specific DEEHIB remains constant.’ We could not figure out, in our copy of the paper, how this number (2268 kg) was obtained.

In order to test Tøien *et al*. hypothesis of storage, we computed savings of hibernation (as percentages) using Tøien *et al*. dataset, as savings = [BMR(empirical)-DEE_H_]/BMR(empirical). This new variable was included as a last column in the spreadsheet of electronic supplementary material and is generated by our script as a sixth plot. If the hypothesis of storage is supported, the log–log regression between savings and *M*_B_ should give a negative exponent, with a reasonable large *R*^2^ (above 0.9, as for DEE_H_ scaling). Whereas the slope is negative, the adjustment was poor (*R*^2^ = 0.24; intercept = −0.0025 ns; slope = −0.06, electronic supplementary material). As stated in our original paper, we believe these large residual errors are due to the use of mass-specific units in these kind of analyses.

More than a decade ago, several authors gave compelling reasons to stop using mass-specific units in metabolic data [13,14]. There is an enormous bias introduced to the data by dividing a random variable measured with error (metabolic rate) by another random variable also measured with error (body mass). Then, calculating a regression coefficient [=cov(*x*,*y*)/var(*x*)], with the *x*-variable that is already included as a denominator of the *y*-variable, inflates the residual error, making the resulting *R*^2^, meaningless. We are not repeating this discussion here, but we note that the bias of mass-specific units is especially important for point estimations (i.e. calculating M_B_zero). We believe this is the reason why previous authors (including Tøien *et al.* analysis) have found that hibernation becomes inefficient at body sizes of thousands of kg. In this comment, we show that M_B_zero could be set to the range of 300–400 kg, which is still within the size range of adult brown bears. Therefore, we think is interesting to keep asking why bears hibernate (or why natural selection promoted hibernation in such large mammals).

## Data Availability

The data are provided in the electronic supplementary material [15].

## Endnote

^1^ Note that Tøien *et al.* [6] quoted incorrectly our estimation of *M*_Blim_ using DEE, which was 1549.7 kg (and not 155 kg as written). Also, Tøien *et al.* [6] described their log–log regression analysis as ‘going from a slightly positive exponent (1.022) to slightly negative exponent (0.981)’. This number, the slope of the regression equation and the allometric exponent (0.981), is positive. In addition, Tøien *et al*. [6] described the plot of fig. 1 (left) as ‘showing the exponential increase in mass specific BMR’ but the curve shows an exponential decay. Also, in their comment, these authors rely on mass-specific units (see the text) of mlO_2_ g^−1^ h^−1^, which also need to be converted to kJ to be comparable, assuming a given respiratory quotient.
